# Wee1 Rather Than Plk1 Is Inhibited by AZD1775 at Therapeutically Relevant Concentrations

**DOI:** 10.3390/cancers11060819

**Published:** 2019-06-13

**Authors:** Angela Flavia Serpico, Giuseppe D’Alterio, Cinzia Vetrei, Rosa Della Monica, Luca Nardella, Roberta Visconti, Domenico Grieco

**Affiliations:** 1 CEINGE Biotecnologie Avanzate, 80145 Naples, Italy; angelaflavia.serpico@hotmail.it (A.F.S.); giusdalt95@gmail.com (G.D.); c.vetrei@studenti.unina.it (C.V.); rosa_d@hotmail.it (R.D.M.); lucanardella95@gmail.com (L.N.); 2 DMMBM, University of Naples “Federico II”, 80131 Naples, Italy; 3 IEOS, CNR, 80131 Naples, Italy; visconti@unina.it; 4 Department of Pharmacy, University of Naples “Federico II”, 80131 Naples, Italy

**Keywords:** Wee1 inhibitor, Plk1 inhibitor, DNA replication checkpoint, combination therapy, concentration range

## Abstract

Wee1 kinase is an inhibitor of cyclin-dependent kinase (cdk)s, crucial cell cycle progression drivers. By phosphorylating cdk1 at tyrosine 15, Wee1 inhibits activation of cyclin B-cdk1 (Cdk1), preventing cells from entering mitosis with incompletely replicated or damaged DNA. Thus, inhibiting Wee1, alone or in combination with DNA damaging agents, can kill cancer cells by mitotic catastrophe, a tumor suppressive response that follows mitosis onset in the presence of under-replicated or damaged DNA. AZD1775, an orally available Wee1 inhibitor, has entered clinical trials for cancer treatment following this strategy, with promising results. Recently, however, AZD1775 has been shown to inhibit also the polo-like kinase homolog Plk1 in vitro, casting doubts on its mechanism of action. Here we asked whether, in the clinically relevant concentration range, AZD1775 inhibited Wee1 or Plk1 in transformed and non-transformed human cells. We found that in the clinically relevant, nanomolar, concentration range AZD1775 inhibited Wee1 rather than Plk1. In addition, AZD1775 treatment accelerated mitosis onset overriding the DNA replication checkpoint and hastened Plk1-dependent phosphorylation. On the contrary selective Plk1 inhibition exerted opposite effects. Thus, at therapeutic concentrations, AZD1775 inhibited Wee1 rather than Plk1. This information will help to better interpret results obtained by using AZD1775 both in the clinical and experimental settings and provide a stronger rationale for combination therapies.

## 1. Introduction

Wee1 is a crucial cell cycle kinase that inhibits activation of cyclin-dependent kinases by phosphorylating the cyclin-dependent kinase (cdk) moiety at inhibitory sites. In particular, Wee1 is important in controlling the onset of mitosis by phosphorylating cdk1 at tyrosine 15 and inhibiting activation of cyclin B-cdk1 (Cdk1), the major M-phase promoting cdk [1]. Cell cycle checkpoints that ensure that cells enter mitosis only after completion of DNA replication (DNA Replication Checkpoint; DRC) or delay mitosis onset in case of DNA damage (DNA Damage Checkpoint; DDC), rely on the action of Wee1 to inhibit Cdk1 activation and the onset of mitosis before DNA replication completion or DNA damage repair [2]. If these checkpoints fail, cells enter mitosis with incompletely or damaged DNA. These conditions result in aberrant mitosis that often ends up in cell death or senescence due to a still poorly defined pathway called mitotic catastrophe. DNA damaging anticancer drugs likely exploit mitotic catastrophe to exert their therapeutic action [2,3]. Thus, cancer cell DDR may oppose therapeutic efficiency of DNA damaging anticancer drugs. These observations have led to the hypothesis that overriding cancer cell DDR would increase mitotic catastrophe, thus, the efficacy of DNA damaging drugs [3]. Following this rationale, an orally available Wee1 inhibitor, AZD1775 (previously named MK1775), has been recently developed and tested in clinical trials in combination with DNA damaging anticancer drugs, with the intent of increasing efficacy [4].

The initial observations appear promising and, although inhibiting Wee1 could have genome-destabilizing effects, clinical trials in which AZD1775 is also used as monotherapy have been initiated, following the idea that overriding the DNA replication checkpoint would also promote mitotic catastrophe [5,6,7,8]. In addition, AZD1775 has also been used in a variety of in vitro studies to assess Wee1 function [9,10,11]. While AZD1775 potently inhibits Wee1, very recently this drug has been demonstrated to inhibit also Plk1 with equal potency to Wee1, in vitro [12]. This observation casts doubts on the interpretation of the mechanism of action of AZD1775 both in the clinical and experimental settings. Thus, we set out to determine whether, at concentrations that have shown therapeutic potential in patients, AZD1775 would elicit effects that are to be ascribed to inhibition of Wee1 or of Plk1 in cancerous and non-cancerous human cell cultures [5,6,7,8]. Our data strongly indicate that at therapeutic concentrations, that span the nanomolar concentration range, the in vivo effects of AZD1775 has to be ascribed to inhibition of Wee1 rather than of Plk1 [5,6,7,8]. This information will provide useful means to interpret experimental as well as clinical data to further develop anticancer strategies.

## 2. Results

### 2.1. Effects on Cdc25C Mobility upon In Vivo Treatment of Different Human Cell Lines with Various Concentrations of BI6727 or AZD1775

To assess whether and at which concentration AZD1775 inhibited Plk1 in vivo, first we used changes in mobility on SDS/PAGE of Cdc25C, the phosphatase that reverses Cdk1 inhibitory phosphorylation at tyrosine 15 (p-Y15-Cdk1) at mitosis onset, upon treatment of mitotic human cells in culture with various concentrations of AZD1775 as readout for Plk1 inhibition. Indeed, Cdc25C is phosphorylated by mitotic kinases including Plk1 [13]. Therefore, the mitotic Cdc25C form undergoes dramatic retardation in mobility, when separated on SDS/PAGE, compared to the interphase form. Indeed, Plk1 inhibition in mitotic cells induces dephosphorylation of Cdc25C that is detectable by a significant increase in Cdc25C mobility on SDS/PAGE [13]. Four different human cell lines were arrested in mitosis by treatment with the microtubule polymerization inhibitor nocodazole: the chromosomally unstable, p53-deficient, cervix cancer HeLa cells, the chromosomally stable, p53-proficient, colon cancer HCT116 cells, the lung adenocarcinoma, p53-proficient, A549 cells and the non-cancerous telomerase-immortalized retinal epithelium, p53-proficient, hTERT-RPE1 cells. Nocodazole-treatment activates the spindle assembly checkpoint, a mechanism that delays mitosis exit when spindle assembly is impaired [14]. Mitotic arrested cells were collected and first treated with various concentrations of the specific Plk1 inhibitor BI6727, from 5 to 100 nM, for 60 min, in the presence of the proteasome inhibitor MG132 (10 µM) to prevent possible cyclin B degradation and mitosis exit, to determine whether Plk1 inhibition effectively affected Cdc25C mobility on SDS/PAGE by immunoblotting (Ib).

**Figure 1:**
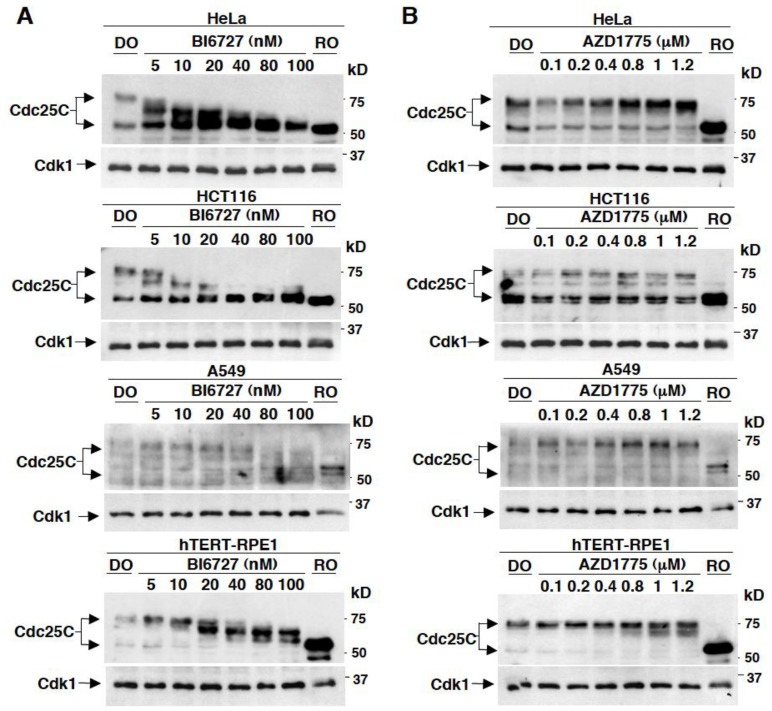
Effects on Cdc25C mobility in different mitotic human cells treated with various concentrations of BI6727 or AZD1775. HeLa, HCT116, A549 and hTERT-RPE1 cells were arrested at prometaphase mitosis by treatment with nocodazole. Prometaphase arrested cell were collected, then the proteasome inhibitor MG132 (10 µM) was added, to prevent possible cyclin B degradation and mitosis exit, and cells split into various samples. Cell samples were further incubated for 60 min with either just dimethylsulfoxide (DO) vehicle, as control and (**A**) with the indicated concentrations of BI6727 (nM); (**B**) with the indicated concentrations of AZD1775 (µM). After incubation, cells were lysed and proteins were separated on SDS/PAGE, blotted and blots probed for Cdc25C and total cyclin B-cdk1 (Cdk1). Data shown are representative of three independent experiments showing similar results.

In all cell lines tested, the Plk1 inhibitor induced dephosphorylation of Cdc25C already at the concentration of 5 nM, that was progressively more marked at increasing concentrations, shown by the increased Cdc25C mobility (Figure 1A; cells were also treated with high doses of RO3306 (10 µM), a rather selective Cdk1 inhibitor, to induce complete mitosis exit and revert Cdc25C to interphase mobility; RO). When used in patients, the effective plasma concentration of AZD1775 has been estimated to be 240 nM [5,6,7]. When mitotic arrested cells were treated under similar conditions with various concentrations, from 100 to 1200 nM, of AZD1775 the mobility of Cdc25C was hardly affected and a slight increase in migration was only detected at 800 nM or above (Figure 1B). Thus, in the therapeutically effective, nanomolar, range AZD1775 does not appear to significantly inhibit Plk1 activity, at least judged by the Plk1-dependent phosphorylation status of Cdc25C (Figure 1A,B).

### 2.2. AZD1775 Potently Inhibits Y15-Cdk1 Phosphorylation at Concentrations that Leave Plk1-Dependent Phosphorylation of Cdc25C Unaffected

**Figure 2:**
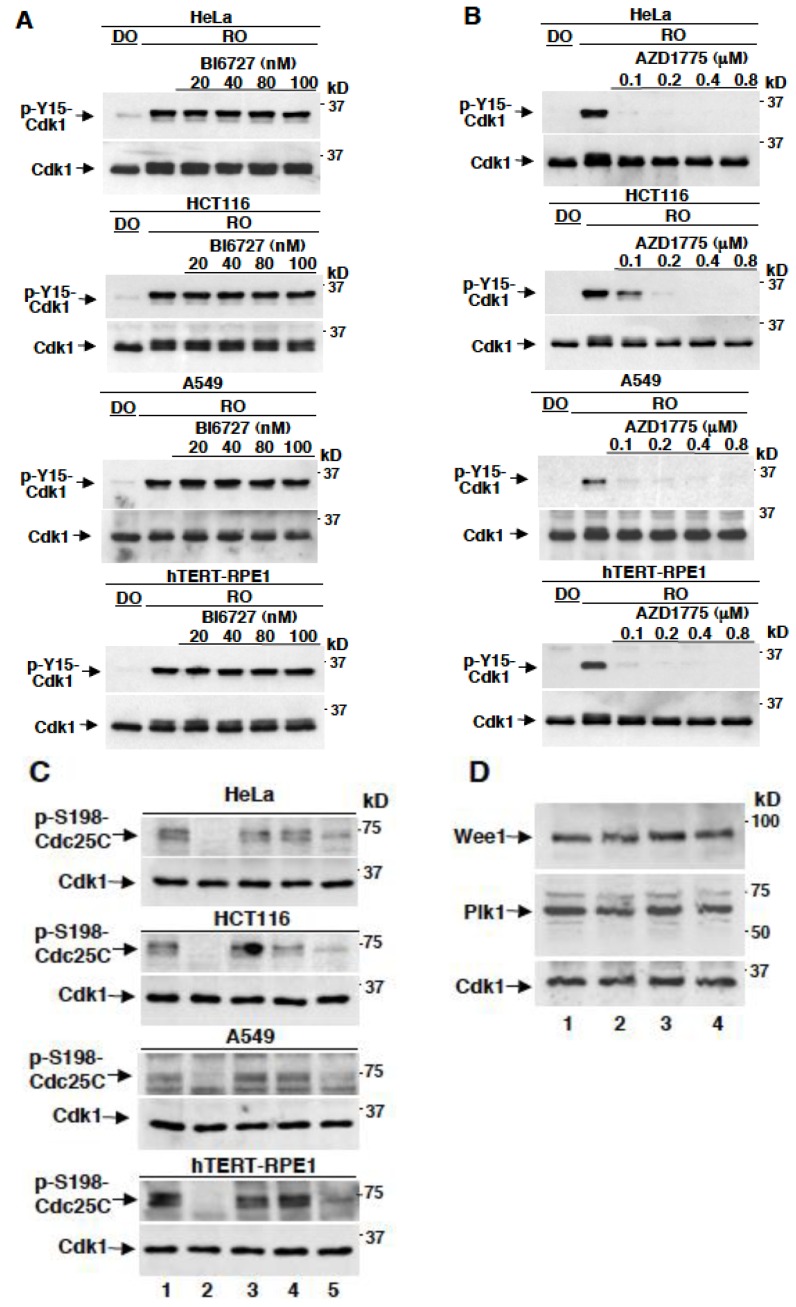
Effects on Y15-Cdk1 phosphorylation in different mitotic human cells treated with various concentrations of BI6727 or AZD1775. Cells were treated with nocodazole as in Figure 1. Prometaphase arrested cells were collected, then the proteasome inhibitor MG132 (10 µM) was added, and cells split into various samples. Cell samples were further incubated for 60 min with either just dimethylsulfoxide (DO) vehicle, as control and (**A**) RO3306 (10 µM) alone or along with the indicated concentrations of BI6727 (nM), or (**B**) RO3306 (10 µM) alone or along with the indicated concentrations of AZD1775 (µM). (**C**) Prometaphase arrested cells were collected, then the proteasome inhibitor MG132 (10 µM) was added, and cells split into various samples. Cell samples were treated with DO (lane 1), BI6727 at 80 nM (lane 2) and AZD1775 at 0.4, 0.8 and 1.6 µM (lane 3, 4, 5, respectively) for further 60 min of incubation. (**D**) Prometaphase arrested HeLa (lane 1), HCT116 (lane 2), A549 (lane 3) and hTERT-RPE1 (lane 4) cells were collected. Cell samples were lysed, and proteins were separated on SDS/PAGE, blotted and blots probed for the indicated antigens. Data shown are representative of three independent experiments showing similar results.

We wanted to ensure that AZD1775 was effectively inhibiting Wee1 under our experimental conditions. To this end, we took advantage of the fact that in mitotic arrested cells Cdk1 maintains its own activation state by inhibiting Wee1. Thus, the levels of p-Y15-Cdk1 are very low in mitotic arrested cells, however, the addition of the potent Cdk1 chemical inhibitor RO3306 (10 µM) to mitotic cells, by de-repressing Wee1 activity, leads to strong phosphorylation of Y15-Cdk1, provided that cyclin B degradation is prevented [15]. Thus, nocodazole-treated, mitotic arrested, HeLa, HCT116, A549 and hTERT-RPE1 cells were further treated, in the presence of MG132 (10 µM), for 60 min either with just dimethylsulfoxide (DO) or with RO3306 (10 µM), in the absence or presence of various doses of BI6727 or AZD1775 (Figure 2A,B). In all cell types, RO3306 induced potent Y15-Cdk1 phosphorylation, by reactivating Wee1, however, while BI6727 did not significantly affect it even at 100 nM, Y15-Cdk1 phosphorylation was strongly inhibited by AZD1775 already at 100 nM, the lowest concentration used (Figure 2A,B). We also assessed the effects of AZD1775 on the specific Plk1-dependent phosphorylation of Cdc25C at serine 198 (p-S198-Cdc25C), detected through a phospho-specific antibody, by treating mitotic arrested cells with BI6727 at 80 nM or with AZD1775 at 0.4, 0.8 and 1.6 µM for 60 min and found that, in all cell lines tested, while treatment with BI6727 completely wiped out the signal, treatment with AZD1775 only partly reduced the signal when added at the highest concentration (1.6 µM; Figure 2C) [16]. All cell lines tested had comparable levels of Plk1 and Wee1 in mitosis, when compared to the levels of expression of Cdk1 (Figure 2D). Together, these data indicate that, when assessed in vivo, AZD1775 potently inhibits Wee1 rather than Plk1 within the therapeutically relevant, nanomolar, concentration range.

### 2.3. AZD1775 but Not BI6727 Induces Premature Onset of Mitosis

We asked whether the fact that at therapeutically relevant concentrations AZD1775 inhibited Wee1 rather than Plk1 in cells was so relevant by exerting biological responses that were to be genuinely ascribed to the inhibition of Wee1 rather than Plk1. Cell cycle progression is monitored by checkpoints, in particular, during DNA replication premature onset of mitosis is prevented by a checkpoint that strongly relies on Wee1 action to keep Cdk1 inactive, and to prevent mitosis onset until completion of DNA replication [2]. On the other hand, Plk1 is important for the resumption of mitosis upon completion of DNA replication and resolution of the DNA damage checkpoint since it has been shown to phosphorylate Wee1 and Cdc25C, promoting their inhibition and activation, respectively [16,17]. Therefore, inhibition of Wee1 or of Plk1 should have clearly opposite effects on mitosis onset, inhibition of Wee1 should accelerate it while inhibition of Plk1 should delay it. Thus, we tested the effect of AZD1775 or of BI6727 on the timing of mitosis onset in HeLa and hTERT-RPE1 cells released from a DNA replication checkpoint, induced by a double thymidine block.

**Figure 3:**
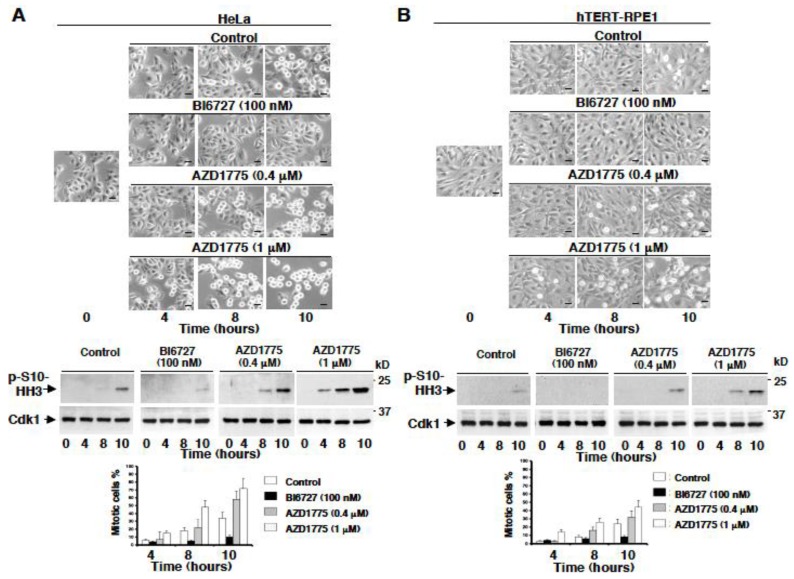
Premature mitosis onset is induced in vivo by treatment with AZD1775 but not with BI6727, morphological analysis. (**A**) HeLa cells and (**B)** hTERT-RPE1 cells were arrested at G1 by a double thymidine block as described in the method section. Cells were released from the block into fresh medium, then, either vehicle (Control) or the indicated concentrations of BI6727 (nM) or AZD1775 (µM) were added and cell samples further incubated for the indicated time points. Photographs were taken at the indicated time points of further incubation (scale bars, 15 µm). Cell samples were also taken, lysed and proteins were separated on SDS/PAGE, blotted and blots probed for the mitotic marker histone H3 phosphorylated at serine 10 (p-S10-HH3) and Cdk1. The graphs show the percent of mitotic cells at the indicated time of incubation. Data shown are representative of two independent experiments showing similar results.

Cells were released from a double thymidine block just in fresh medium plus dimethylsulfoxide vehicle (Control) or in fresh medium containing BI6727 (100 nM), or in fresh medium containing AZD1775 at 0.4 µM or at 1 µM, and further incubated, then, cell samples were imaged and taken at the indicated time points (Figure 3). Control cells started to enter mitosis by approximately 8–10 h from the release as shown by their rounding up and partial detachment from the bottom of the tissue culture dish as well as by the increase in phosphorylation of histone H3 at serine 10 (p-S10-HH3), a mitotic marker (Figure 3; Control; the larger number of mitotic HeLa relatively to hTERT-RPE1 cells at the 10 h time point reflect the fact that HeLa has an overall shorter cell cycle timing compared to hTERT-RPE1 cells). In both cell lines, cells remained rather flat showing little rounding up until 10 h and very low p-S10-HH3 levels in the presence of BI6727 (Figure 3, BI6727 100 nM). Treatment with AZD1775, instead, induced a dose-dependent acceleration of mitosis onset as judged by the shortened time by which cells rounded up and accelerated the appearance of p-S10-HH3 (Figure 3; AZD1775 0.4 µM and 1 µM).

**Figure 4:**
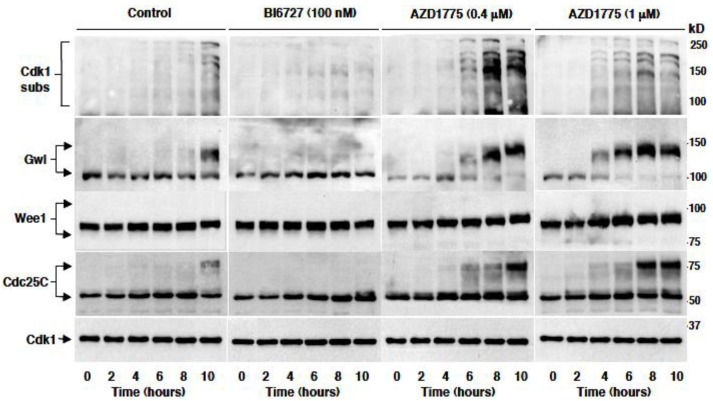
Premature mitosis onset is induced in vivo by treatment with AZD1775 but not with BI6727, biochemical analysis. HeLa cells were arrested at G1 by a double thymidine block as described in the method section. Cells were released from the block into fresh medium, then either vehicle (Control) or the indicated concentrations of BI6727 (nM) or AZD1775 (µM) were added and cells further incubated. Cell samples were taken at the indicated time points of further incubation and lysed. Lysate proteins were separated on SDS/PAGE, blotted and blots probed for the indicated antigens. Data shown are representative of four independent experiments showing similar results.

To better biochemically monitor the timing of mitosis onset, HeLa cells were treated as indicated for the experiment described for Figure 3 and sampled at the indicated time points from the release from a double thymidine block in the various conditions (Figure 4). Cell lysates were probed for an antibody recognizing phosphorylated Cdk1 substrates (Cdk1 subs) as well as for Greatwall kinase (Gwl), Wee1 and Cdc25C, proteins that undergo a substantial, phosphorylation-dependent, mobility shift on SDS/PAGE at the onset of mitosis. Control cells started entering mitosis at around 8 h from the release and about 40% were in mitosis by 10 h, as shown by increased Cdk1 phospho-substrate signal and by the retardation of Gwl, Wee1 and Cdc25C mobility (Figure 4; Control). Cells treated with BI6727 were, instead, strongly delayed in mitosis onset as shown by the lack of substantial increase in Cdk1 phospho-substrate signal and of Gwl, Wee1 and Cdc25C mobility retardation even at the 10 h time point (Figure 4; BI6727 100 nM). Treatment with AZD1775, on the contrary, significantly accelerated mitosis onset in a dose-dependent manner (Figure 4; AZD1775 0.4 µM and 1 µM).

### 2.4. AZD1775 Hastens Plk1-Dependent Phosphorylation In Vivo

**Figure 5:**
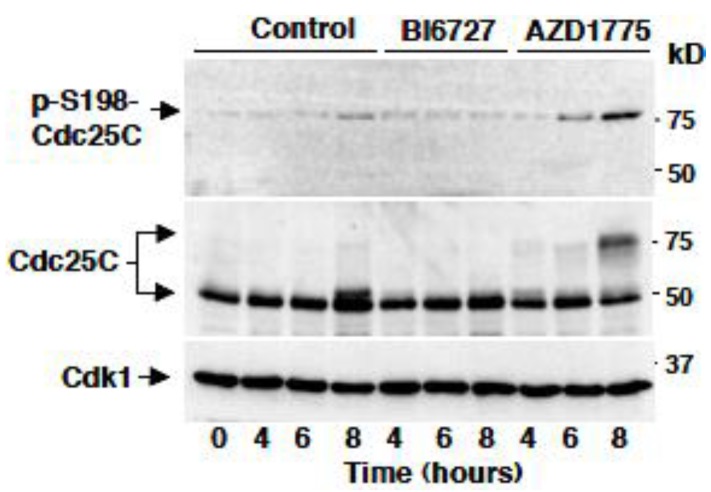
AZD1775 hastens Plk1-dependent phosphorylation in vivo. Cells were released from the block into fresh medium, then either vehicle (Control) or BI6727 (100 nM) or AZD1775 (1 µM) were added. Cell samples were taken at the indicated time points of further incubation and lysed. Lysate proteins were separated on SDS/PAGE, blotted and blots probed for the indicated antigens. Data shown are representative of three independent experiments showing similar results.

At the onset of mitosis, Plk1 has been shown to directly phosphorylate Cdc25C at serine 198 [16]. We, therefore, asked whether AZD1775 affected Plk1-dependent S198-Cdc25C phosphorylation. To this end, HeLa cells were released from a double thymidine block just in fresh medium plus dimethylsulfoxide vehicle (Control) or in fresh medium containing BI6727 (100 nM), or in fresh medium containing AZD1775 (1 µM), sampled at the indicated time points and phosphorylated S198-Cdc25C was detected (Figure 5; p-S198-Cdc25C; total Cdc25C were also detected from the same samples). The data show that in control cells little signal of the Plk1-dependent phosphorylation of Cdc25C was detected at the 8 h time point, as cells were starting to enter mitosis. The signals remained at baseline in the BI6727-treated cell samples, while treatment with AZD1775 substantially accelerated the appearance of the Plk1-dependent phosphorylation. Thus, treatment with AZD1775 not only did not inhibit Plk1-dependent phosphorylation but actually hastened it, likely as a consequence of accelerated Cdk1 activation and mitosis onset in the absence of substantial inhibition of Plk1 activity (Figure 5).

## 3. Discussion

Wee1 is a crucial inhibitor of Cdk1 activation [1,2]. Cells rely on Wee1 activity to implement important checkpoints ensuring that mitosis onset is delayed until completion of DNA replication or in case of DNA damage [2,3,4]. On the other hand, Plk1 is important for the timely resumption of mitosis upon silencing of these checkpoints [12,16]. The evidence that overriding the DRC or DDC induces cells to die upon the following mitosis, through mitotic catastrophe, has suggested that inhibiting functions that sustain DRC or DDC, like that of Wee1, could be beneficial for cancer therapy alone, by overriding the DRC, or in combination with DNA damaging antineoplastic drugs, by overriding the DDC [2,3]. AZD1775, an orally available Wee1 inhibitor, was developed for these purposes and is currently being tested in cancer patients, alone or in combination therapies [4,5,6,7,8]. The initial data appear promising in terms of efficacy and tolerability [5,6,7,8]. On the other hand, initial trials in patients with Plk1 inhibitors have been disappointing [18]. Recently, it has been shown that AZD1775 can bind and inhibit Plk1 in vitro [12]. These observations have suggested that the in vivo effects of AZD1775, both in the clinical and in the experimental setting, might be due to the inhibition of both Wee1 and Plk1 [12,19].

The data we gathered indicate instead, that, when added in the nanomolar concentration range, the effects of AZD1775 in human cells are to be ascribed to inhibition of Wee1 rather than of Plk1. In that concentration range, AZD1775 does not inhibit phosphorylation of direct Plk1 substrates but, on the contrary, this drug is able to accelerate mitosis onset by overriding the DRC. Conversely, Plk1 inhibition delays mitotic resumption upon DRC resolution. Moreover, by promoting DRC override and accelerating the onset of mitosis, AZD1775 actually hastens Plk1-dependent phosphorylation. These observations have importance in the interpretation of clinical effects of AZD1775 since pharmacokinetic and pharmacodynamic studies in patients have established that this drug is efficient at plasma concentrations that remain within the nanomolar range. Thus, the beneficial effects in cancer patients of AZD1775 can be mechanistically interpreted as due to interference with the function of Wee1 rather than of Plk1. In addition, our observations will help to establish a more straightforward data interpretation when AZD1775 is used in the nanomolar concentration range in experimental settings.

## 4. Materials and Methods

### 4.1. Cell Lines and Cell Culture

HeLa and HCT116 cells were grown in Dulbecco’s modified Eagle’s medium (DMEM) (Sigma-Aldrich, St. Louis, MO, USA), A549 cells were grown in RPMI 1640 medium (Sigma-Aldrich), HTERT-RPE1 cells were grown in DMEM/F12 medium (Gibco, Thermo Fisher Scientific, Waltham, MA USA), all media supplemented with 10% fetal bovine serum (FBS; GE Healthcare Life Sciences, Pittsburgh, PA, USA), 1% penicillin/streptomycin (Euroclone, Pero, MI, Italy) and incubated in a humidified incubator at 37 °C with 5% CO_2_.

### 4.2. Cell Treatments and Reagents

DNA replication checkpoint-arrested cells were obtained by a double thymidine block [20]. Cells were incubated with 4 mM thymidine (Sigma-Aldrich) for 18 h, released by rinsing thoroughly with phosphate-buffered saline (PBS) (Euroclone) and incubated in fresh medium. After 6 h incubation, cells were blocked again with 4 mM thymidine for 18 h. Cells were washed twice with PBS and once with fresh medium before incubation into fresh medium, containing dimethylsulfoxide (Sigma-Aldrich; control cells), AZD1775 (previously named MK-1775; Selleckchem, Houston, TX, USA) or BI-6727 (Selleckchem) at the indicated concentrations. Cell samples were then taken at the indicated time points, washed once in PBS and lysed with 5 volumes of NP-40/EB lysis buffer (0.2% NP-40; 80 mM 2-glycerophosphate, 10 mM MgCl_2_ and 20 mM EGTA; Sigma-Aldrich). Lysates were incubated 30 min on ice and then spun for 20 min at 13,200 rpm in a refrigerated microfuge (4 °C). Prometaphase-arrested cells were obtained by performing a double thymidine block, as described above, followed by a release into fresh medium containing nocodazole (Calbiochem, Merck Millipore, Billerica, MA, USA; used at 100 ng/mL for HeLa and HCT116 cells and at 1 μg/mL for A549 and HTERT-RPE1 cells) for 12 (HeLa, HCT116 and A549 cells) or 16 h (HTERT-RPE1 cells). Cells that detached from substrate were recovered by shake-off, collected and plated into new dishes. Then, synchronized cells were exposed for an hour to either AZD1775 or BI6727 for an hour in the presence of MG132 (Calbiochem) at 10 μM and RO3306 (Calbiochem) at 10 μM.

### 4.3. Immunoblotting Analysis

After drug treatments, as described in figure legends, cells were lysed as described above (cell treatments and reagents). Total cell lysates were subjected to SDS-PAGE and blotted onto nitrocellulose membrane (GE Healthcare Life Sciences). Membranes were blocked with 5% not fat dry milk (NFDM; BioRad, MI, Italy) in PBS plus Tween 0.02% (TPBS) or tris-buffered saline (TBS) plus Tween 0.02% (TTBS) for 1 h and then incubated with primary antibodies (1:1000 diluted in TPBS or TTBS) overnight at 4 °C. After washing twice with buffer, the filters were incubated with secondary peroxidase-conjugated antibodies for an hour at room temperature. Detection was performed using an Enhanced ChemiLuminescence (ECL) kit (GE Healthcare Life Sciences).

### 4.4. Antibodies

Antibodies against Cdc25C were from Cell Signaling Technologies (#4688S, Danvers, MA, USA) and Santa Cruz Biotechnology (#SC-327, Dallas, TX, USA). Antibodies against P-Cdc25C (S198), Wee1, P-Cdk1 (Y15), P-MAPK/CDK-substrates (PXS*P and/or S*PXR/K) and P-HH3 (S10) were from Cell Signaling Technologies (#9529S; #13084S; #9111S; #2325S; #9701S respectively). Antibodies against P-Wee1 (Ser53) were from Bioss (#bs-5589R, Woburn, Massachusetts, USA). Antibodies against Cdk1 were from Santa Cruz Biotechnology (#sc-54). Antibodies against MASTL were from Abcam (#ab86387, Cambridge Science Park, Cambridge, UK).

### 4.5. Microscopy

Cells were synchronized at early S-phase by a double thymidine block and then incubated with different chemicals (as previously described) upon release from the block. Cells were photographed in phase contrast at indicated time points (LEICA DFC320) and mitotic cells were visually scored.

## 5. Conclusions

AZD1775 is in clinical trials as a single anticancer treatment or in combination with DNA damaging drugs by its ability to override DRC and DDC, given its potent activity as a Wee1 inhibitor. AZD1775 has been recently shown to inhibit Plk1 with equal potency to Wee1 in vitro. We have tested the effect of AZD1775 in human cell culture in vivo and concluded that, in the nanomolar concentration range, AZD1775 inhibits the action of Wee1 but not that of Plk1. Since AZD1775 has been shown to exert beneficial effects as an anticancer drug at plasma concentrations in patients within the nanomolar concentration range, our data indicate that the effects of AZD1775 should be mechanistically attributed to inhibition of Wee1 rather than inhibition of Plk1. This information will help to strengthen the rationale for combination therapies, in which Wee1 inhibition will be instrumental. In addition, our data may facilitate the interpretation of experimental data derived from studies using AZD1775.
